# Underwater Invisible Light Communication Network with Beam Steering Technology for Dynamic Switching Between Aerial and Underwater Optical Paths

**DOI:** 10.3390/s25041053

**Published:** 2025-02-10

**Authors:** Kiichiro Kuwahara, Keita Tanaka, Ayumu Kariya, Shogo Hayashida, Takahiro Kodama

**Affiliations:** 1 Faculty of Engineering and Design, Kagawa University, 2217-20 Hayashi-cho, Takamatsu 761-0396, Kagawa, Japan; 2 LED Backhaul Project, Sangikyo Corporation, 4509 Ikebe-machi, Tsuzuki-ku, Yokohama-shi 224-0053, Kanagawa, Japan; tanakakei@sangikyo.co.jp (K.T.); hayashidas@sangikyo.co.jp (S.H.)

**Keywords:** optical wireless communication, optical path switching, aerial and underwater channel

## Abstract

This study proposes a bidirectional underwater optical wireless communication network that maximizes data transmission capacity by dynamically switching between underwater and aerial optical paths based on channel conditions. The proposed system employs adaptive modulation and beam steering techniques to address dynamic factors, such as turbidity and transmission distance, in underwater channels. The experimental results revealed that switching to the aerial optical path when the underwater transmission distance exceeded 1.8 m led to significant performance improvements, with consistent SNR and bit rates maintained in the aerial channel, unlike the exponential degradation observed underwater. Dynamic evaluations demonstrated that the system maintained high transmission capacity and SNR stability, even with incremental increases in underwater distances. In a 4K UHD video streaming experiment, switching from the underwater optical path to the aerial path reduced video quality degradation, delivering near-original video quality with latency as low as 20 ms. Furthermore, tolerance experiments for beam steering misalignment showed a sharp performance drop at a maximum misalignment of 2 degrees, with a 12 dB SNR loss and a reduction of 222 Mbps in transmission capacity. These findings suggest that selectively utilizing underwater and aerial optical paths based on channel conditions enables reliable and efficient data transmission, paving the way for next-generation underwater optical wireless communication networks.

## 1. Introduction

The next-generation mobile communication standard, 6G, aims to enhance communication speed, ultra-low latency, and large-scale device connectivity. To achieve these goals, integrating non-terrestrial networks (NTNs) with traditional terrestrial communication networks has emerged as a key enabler. By leveraging diverse NTN environments—including satellite communications, high-altitude platforms (HAPSs), drone communications, and underwater communications—it is possible to build a robust next-generation communication infrastructure. This infrastructure will provide seamless connectivity from urban areas to remote regions, oceans, and outer space [1,2,3,4,5,6].

Sustainable communication technologies that reduce environmental impact and support real-time, high-precision data transmission are fundamental to 6G. In this context, the multi-layered network architecture of NTNs is crucial. For example, satellite communications and drones can enhance terrestrial networks for high-density urban areas, improving robustness and flexibility [7,8,9,10]. Additionally, communication in unique environments, such as shallow and deep seas, requires breakthroughs beyond conventional radio communication technologies regarding range and bandwidth. Optical wireless communication (OWC) has attracted significant attention for addressing these challenges [11,12,13,14,15].

OWC offers a broader bandwidth than radio frequency communication, avoiding frequency band congestion, and it is suitable for terrestrial and non-terrestrial scenarios [16]. Beam steering technology, a core component of OWC, enables high-precision and dynamic communication links [17,18,19].

Underwater optical wireless communication (UWOC), a specialized subset of OWC, faces challenges due to turbidity and transmission distance dependency. These factors cause significant signal strength fluctuations, making adaptive modulation techniques based on the signal-to-noise ratio (SNR) highly effective for ensuring stable communication [20,21,22]. In underwater environments, visible light in the 450–500 nm range exhibits minimal loss and is suitable for long-distance transmission [23]. For shallow waters with significant human activity, wavelengths beyond 830 nm, particularly infrared light at 850 nm, provide high confidentiality and adaptive modulation capabilities [24,25]. Despite these advantages, high turbidity or variable transmission distances may lead to communication outages, even with the most straightforward modulation schemes.

Recent studies have demonstrated the potential of optical wireless communication between underwater and aerial drones, leveraging the relatively stable aerial channel to address the limitations of standalone underwater channels [26]. This approach, which actively leverages the aerial channel, offers a promising pathway toward constructing optical wireless networks with greater robustness against environmental variations than standalone underwater channels.

This paper proposes an optical wireless network capable of seamlessly switching between underwater and aerial optical paths based on underwater channel turbidity and transmission distance, thereby maximizing transmission capacity. Specifically, we developed a technique to dynamically establish an aerial optical path by deploying relay nodes equipped with large acrylic mirrors and appropriately configuring the optical transceivers’ beam steering and receiving angles. The system achieves highly accurate and stable communication even in dynamic underwater environments by combining adaptive modulation with optical path switching. This paper expands on our previous work in [27], demonstrating a static performance evaluation and 4K video transmission experiment for a mobile optical wireless network architecture consisting of two underwater optical wireless transceivers and an aerial relay node deployed in shallow waters. Newly added contributions include a model for calculating underwater transmission distances and propagation delays based on beam steering angles and evaluations of dynamic performance and alignment tolerance in underwater and aerial optical paths.

The paper is structured as follows. Section 2 discusses the related work. Section 3 describes the system architecture and key components, comparing O/E/O and mirror-based relays. Section 4 provides details of the experimental setup and the signal processing techniques employed for underwater and aerial optical wireless communication. Section 5 presents the evaluation results of SNR and transmission capacity, comparing performance in underwater and air optical paths under various environmental conditions. Section 6 demonstrates the system’s capability through a 4K UHD video transmission experiment, validating its performance in shallow seawater scenarios. Section 7 evaluates the system’s tolerance to angular misalignment in the beam steering mechanism, providing insights into practical deployment. Section 8 concludes this study, highlighting the system’s feasibility, effectiveness, and potential for future underwater and aerial optical communication advancements.

## 2. Related Works

Numerous studies have explored optical wireless communication (OWC) technologies for terrestrial and non-terrestrial applications. The broader bandwidth and reduced frequency congestion offered by OWC have positioned it as a promising solution for the high-speed, high-capacity demands of 6G [11,12,13,14,15]. Beam steering technology has been highlighted as a critical component of OWC, enabling the dynamic establishment of high-precision communication links in terrestrial and non-terrestrial scenarios [17,18,19]. However, integrating underwater optical wireless communication (UWOC) with aerial optical networks remains a relatively unexplored field.

In underwater communication, studies have shown that visible light within the 450–500 nm range minimizes loss, making it suitable for long-distance transmission in clear water [23]. Real-time communication has also been successfully achieved in visible light underwater optical wireless communication systems [28].

For shallow waters with significant human activity, infrared wavelengths, such as 850 nm, provide enhanced confidentiality and adaptability [24,25]. Despite these advancements, the challenges posed by high turbidity and fluctuating transmission distances remain significant barriers to achieving stable UWOC [29]. To address these issues, recent research has investigated the use of various modulation schemes tailored to dynamic underwater channels, aiming to enhance communication performance under rapidly changing conditions [30].

Recent research has investigated the use of aerial platforms to address these challenges. For instance, ref. [26] demonstrated optical wireless communication between underwater and aerial drones, leveraging the aerial channel’s relative stability to improve overall communication robustness. While this approach shows promise, it has not been optimized for dynamic environments where underwater conditions fluctuate rapidly.

Compared to prior works, this paper advances the state of the art by introducing a hybrid optical wireless network that seamlessly switches between underwater and aerial optical paths. This network integrates adaptive modulation techniques to counteract signal fluctuations and deploys relay nodes with large acrylic mirrors for dynamic optical path configuration. Additionally, this study introduces a new model for calculating underwater transmission distances and propagation delays based on beam steering angles. This work evaluates dynamic performance and alignment tolerance and provides a comprehensive solution for robust optical wireless communication in underwater and aerial environments. Finally, the system’s feasibility is validated through real-world 4K UHD video transmission experiments in shallow seawater scenarios, demonstrating its potential for practical deployment.

## 3. System Configuration and Application

**Figure 1:**
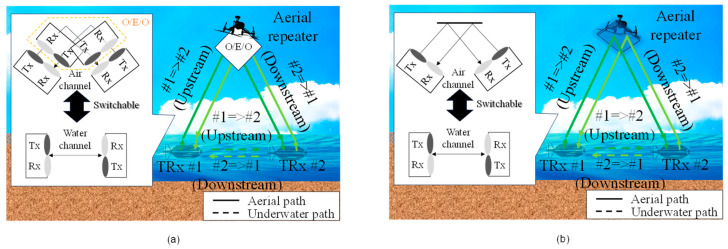
Structure of (**a**) O/E/O relay, (**b**) mirror-based relay.

Figure 1a,b illustrate the architecture of a bidirectional UWOC system capable of switching between underwater and aerial optical paths, with and without optical/electrical/optical (O/E/O) conversion in the aerial optical relay. Latency varies depending on the O/E/O configuration. In O/E/O relays without digital signal processing, the delay caused by analog processing alone is minimal, typically limited to a few microseconds [31]. Consequently, for transmissions over several meters, both O/E/O relays and mirror-based relays are dominated by propagation delay, typically on the order of several hundred microseconds. Table 1 summarizes the characteristics of these configurations. The O/E/O relay requires a power supply for active signal processing, whereas the mirror-based relay requires no power supply for communication-related operations. Mirror-based relays outperform O/E/O relays regarding cost, weight, and power consumption. However, O/E/O relays have the advantage of signal regeneration and optical power gain after optical relaying, particularly when the transmission distance of the aerial optical path increases. Nevertheless, the transceivers in the optical relay must be aligned with the steering angles of the underwater optical transceivers, resulting in complex optical axis alignment. Therefore, mirror-based relays are better suited for shallow water environments where underwater drones can maintain sufficient transmission power through aerial channels when positioned near the water surface.

The proposed system assumes that the distance between underwater optical transceivers varies as two underwater mobile terminals move while the aerial relay node adjusts vertically through attitude control. While placing the aerial relay directly on the water surface is theoretically possible and reduces battery usage, maintaining a stable position is challenging due to wave motion. Hence, it is preferable to deploy the relay in the air, where gyroscopes and inertial measurement units can maintain relatively stable positioning despite susceptibility to wind. When placed close to the water surface, the relay must be positioned at a sufficient height to avoid the effects of waves.

The selection of optical paths is based on the underwater channel conditions as follows.

If the transmission distance in the underwater channel is short, the turbidity is low, and if the received SNR is high, the system selects the underwater optical path. During underwater path selection, the two optical transceivers establish bidirectional beam communication while facing each other.

The system switches to the aerial optical path if the transmission distance in the underwater channel is long, the turbidity is high, and the received SNR is low. During aerial path selection, the two underwater optical transceivers direct their beams toward the mirror in the aerial node. To enable seamless path switching through steering angle adjustment alone, the depth of the two underwater drones is kept uniform. Furthermore, to simplify overall control, the mirror surface is fixed horizontally relative to the water surface, avoiding any changes to its angle. The steering angles of the two optical transceivers are adjusted to ensure that the incident and reflection angles on the mirror are equal. The communication direction from transceiver #1 to #2 is upstream, while the opposite direction is downstream.

**Figure 2:**
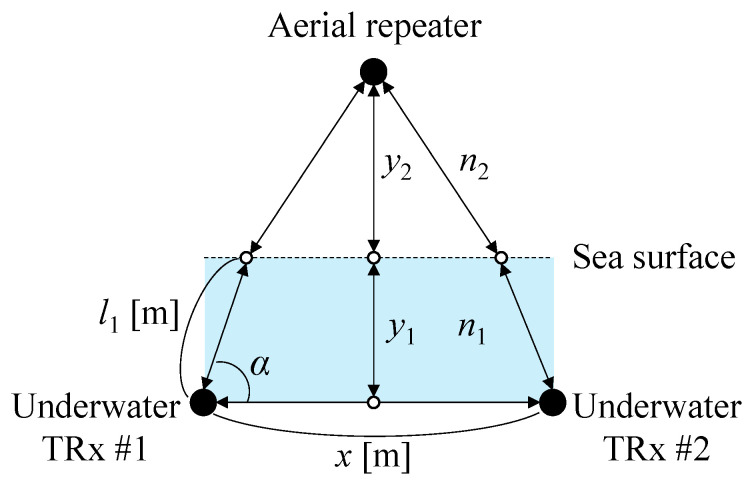
Geometric configuration.

Figure 2 shows the geometric configuration of the system, assuming calm water with no wave-induced variations. The distance between underwater optical transceivers, the water depth at their positions, and the height of the aerial relay node above the water surface are denoted as *x*, *y*_1_, and *y*_2_, respectively. The distances from the underwater optical transceivers to the water surface for the aerial optical path are represented as *l*_1_. The refractive indices of air and water are assumed to be 1 and 1.33, respectively. To maximize the effectiveness of the aerial optical channel relative to the underwater optical channel, *l*_1_ should be minimized. Furthermore, to reduce the operational range of the steering angles, *y*_2_ should also be minimized. The parameter *x* can be expressed using *α* as follows:

(1) $$x=2\left(\frac{y_{1}}{\tan\alpha }+\frac{y_{2}}{\tan\left[\frac{\pi }{2}-\sin^{-1}\left\{\frac{n_{1}}{n_{2}}\sin\left(\frac{\pi }{2}-\alpha \right)\right\}\right]}\right)$$

The underwater channel length *l* in the aerial optical path can be expressed as follows:

(2) $$l=\frac{2y_{1}}{\sin\alpha }$$

The propagation delay times *T*_underwater_ and *T*_aerial_ for the underwater and aerial optical paths, respectively, can be expressed using the speed of light *c* as follows:

(3) $$T_{\mathrm{underwater}}=\frac{n_{1}x}{c}=\frac{2n_{1}}{c}\left(\frac{y_{1}}{\tan\alpha }+\frac{y_{2}}{\tan\left[\frac{\pi }{2}-\sin^{-1}\left\{\frac{n_{1}}{n_{2}}\sin\left(\frac{\pi }{2}-\alpha \right)\right\}\right]}\right)$$

(4) $$T_{\mathrm{aerial}}=\frac{2}{c}\left(\frac{n_{1}y_{1}}{\sin\alpha }+\frac{n_{2}y_{2}}{\sin\left[\frac{\pi }{2}-\sin^{-1}\left\{\frac{n_{1}}{n_{2}}\sin\left(\frac{\pi }{2}-\alpha \right)\right\}\right]}\right)$$

**Figure 3:**
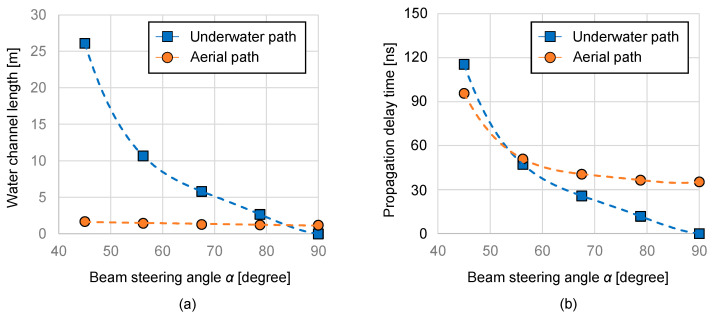
Comparison of (**a**) underwater length, (**b**) propagation delay.

Figure 3a illustrates the underwater and aerial channel distances as a function of α, assuming *y*_1_ = 0.6 m and *y*_2_ = 4.5 m. In the underwater optical path, the channel distance matches the value of *x*. Conversely, in the aerial optical path, the underwater optical path is effective when *x* < 1.2 m, while the aerial optical path is effective when *x* > 1.2 m. Figure 3b shows the propagation delay time for underwater and aerial paths as a function of *α* when *x* > 11 m, where the propagation delay for the aerial optical path becomes shorter than that of the underwater optical path.

**Figure 4:**
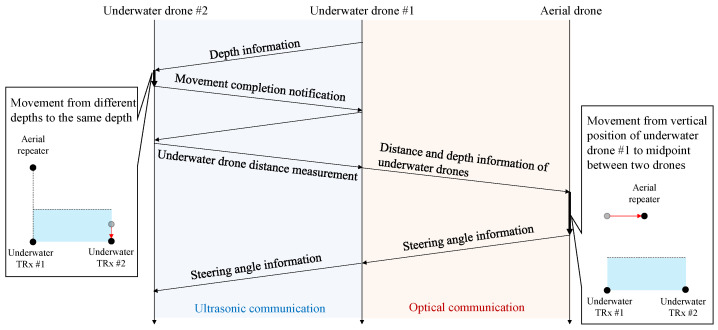
Handshake sequence between the underwater and aerial drone.

Figure 4 illustrates the communication and data exchange process between underwater drone #1, underwater drone #2, and an aerial drone, utilizing ultrasonic and optical wireless communication.

Following the transmission of depth information between underwater drone #1 and underwater drone #2 via ultrasonic communication, a crucial movement completion notification is sent. This notification, also using ultrasonic communication, is a key step in the process, as it confirms the successful execution of a movement. The process continues by calculating the distance between the underwater drones, which is measured and shared through ultrasonic communication. Subsequently, the precision of the distance and depth information is highlighted. It is transmitted between underwater drone #1 and the aerial drone using optical wireless communication, leveraging its high-speed and reliable data transmission capabilities across the water-to-air boundary. For proper navigation and alignment, steering angle information is exchanged in two ways. This dual-mode approach, between the underwater drones via ultrasonic communication and underwater drone #1 and the aerial drone via optical wireless communication, is a significant factor in ensuring accurate and synchronized operations across water and air environments.

Overall, this setup effectively combines the strengths of ultrasonic communication for underwater reliability and optical wireless communication for precise data exchange between the water and air interfaces.

**Figure 5:**
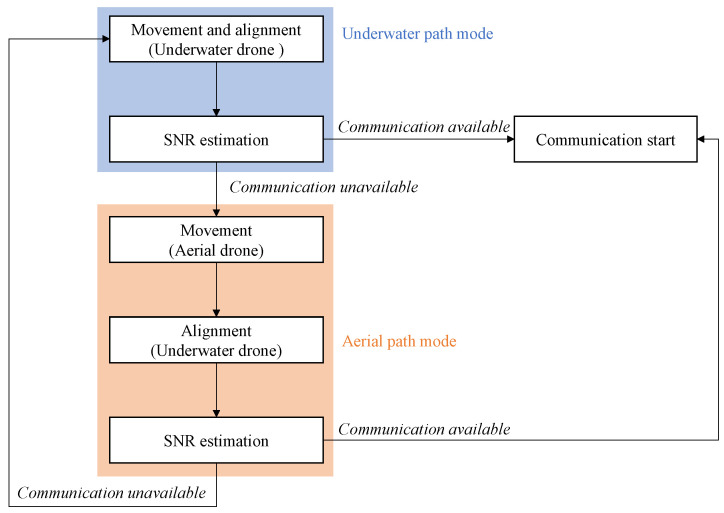
Operation flow of drive and communication systems.

Figure 5 summarizes the operational flow of the drive and communication systems when switching between underwater and aerial optical paths for both underwater and aerial drones. Initially, the underwater drones adjust their positions and alignments. Channel estimation is then performed to measure the received SNR. If the SNR allows communication, the system initiates transmission. The system switches to the aerial optical path if the channel estimation determines that even the lowest-order adaptive modulation is infeasible. The aerial relay node, corresponding to an aerial drone, moves horizontally and vertically. After adjusting the beam steering angles, channel estimation is conducted again. If the lowest-order modulation is feasible, communication begins. If deemed infeasible, the underwater drones adjust their positions vertically or horizontally to improve the received SNR and revert to the underwater optical path, repeating the process until successful communication is achieved.

## 4. Principle Experimental Setup

**Figure 6:**
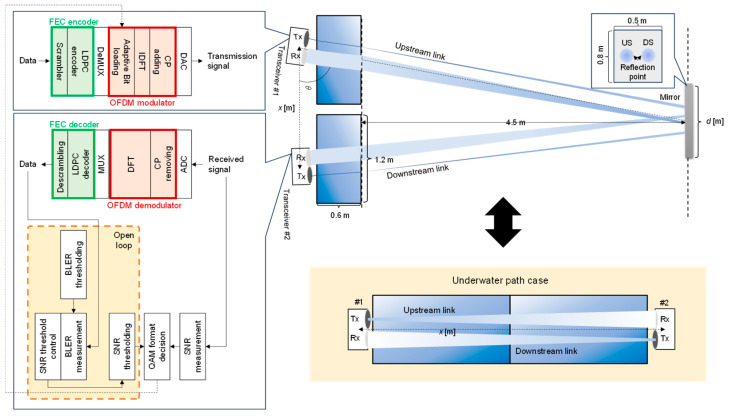
Experimental setup of the aerial and underwater optical path system.

**Figure 7:**
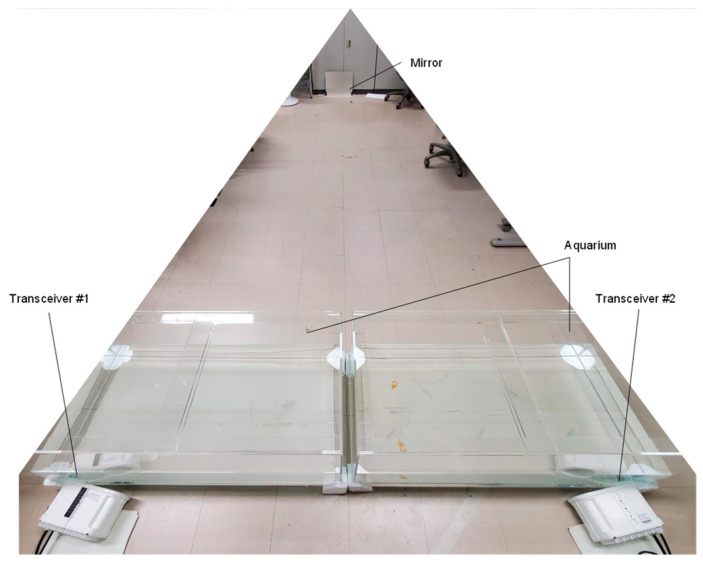
Picture of the aerial optical path system.

Figure 6 illustrates the experimental setup for a full-duplex underwater optical wireless communication system designed to emulate the switching of optical paths between underwater and air. Figure 7 shows the experimental setup under indoor conditions, simulating the air optical path. The upstream and downstream processing procedures are identical; this paper describes the uplink processing as a representative example.

The optical wireless transceiver in this experiment consists of a near-infrared LED with a peak wavelength of 850 nm as the light source. The transmission power is 180 mW at peak and typically 50 mW. The LED has a half-power angle of ±17° without a lens. The system employs a Si PIN photodiode detector on the receiving side with a receiving aperture of 100 mm. The field of view (FOV) is ±0.5° with a lens. The receiver gain is controlled using automatic gain control (AGC).

In this experiment, we employed an optical wireless transceiver based on a light-emitting diode (LED) backhaul compliant with Class 1 eye-safe standards, in line with the standardization of the PHY and MAC layers of IEEE 802.15.13 [32]. On the transmitter side, the forward error correction (FEC) encoding module processes the original data through a scrambler and low-density parity check (LDPC) encoding, generating a bitstream that includes parity bits. A DeMUX converts the bitstream into symbols for each subcarrier. The modulation symbols selected correspond to one of the M values of quadrature amplitude modulation (QAM). The orthogonal frequency division multiplexing (OFDM) signal modulation module dynamically assigns bits to subcarriers based on the SNR. Subcarriers are generated via inverse discrete Fourier transform, and a cyclic prefix (CP) is added to improve resistance to inter-symbol interference. The multiplexed OFDM signal is converted into an analog transmission signal by a digital-to-analog converter. The 850 nm light emitted by the LED passes through underwater and air channels via a water tank.

For the underwater optical path, the transmission distance of the underwater channel is modified by changing the tank’s length, width, or number of traversal stages. For the air optical path, the orientation of the tank and the position of the silvered glass mirror is fixed. At the same time, the transmission distance is varied by adjusting the distance *x* between the optical wireless transceivers and the steering angle α. The water tank is filled with either tap water or seawater with a turbidity of 2.2 nephelometric turbidity units (NTUs). This study measured turbidity using a turbidity meter (KY HOPE, WGZ-1B) based on the principle of light scattering. The unit of turbidity, 1 NTU, is the turbidity of 1 L of purified water containing 1 mg of formazin standard solution [33].

On the receiver side, an optical detector converts the optical signal into an electrical signal, digitized by an analog-to-digital converter. In the OFDM signal demodulation module, the CP is removed, and subcarriers are separated via discrete Fourier transform. Each subcarrier symbol is converted back into a bitstream by a MUX. The FEC decoding module processes the data through LDPC decoding and descrambling, restoring the original data. The maximum transmission capacity in this evaluation corresponds to the capacity at which signal quality is sufficient to allow error correction through adaptive modulation.

The OFDM signal dynamically adjusts the number of bits assigned to each subcarrier based on the received SNR using an adaptive modulation algorithm [34]. This algorithm selects the optimal modulation scheme for each subcarrier based on the SNR and integrates open-loop and closed-loop control mechanisms. Open-loop control adjusts the threshold SNR used as a reference. Specifically, the threshold is increased when the block error rate (BLER) after demodulation is high to ensure communication reliability. Conversely, when the BLER is low, the threshold is decreased to enhance communication efficiency. These adjustments dynamically modify the QAM table, managing the modulation scheme and enabling adaptive responses to varying conditions. The adaptive modulation algorithm also implements a feedback loop in which the transmitter and receiver periodically exchange probe frames. The receiver determines the appropriate QAM format based on the measured SNR and sends feedback messages to the transmitter. Real-time adjustments to the modulation scheme ensure optimal transmission quality. The probe frame exchange follows a predefined modulation scheme and redundancy level, and if the SNR is too low, communication is not established, preventing inefficient transmission attempts. Because BLER measurement requires a specific period, SNR threshold changes are typically gradual. This characteristic allows adaptive modulation to maintain stability while improving performance. The dynamic bit allocation algorithm for the optical transceiver used in this study adheres to ITU-T G.9960 [35] and ITU-T G.9961 [36], which specify the transmitter, receiver, and data link layer requirements for optical wireless communication systems.

In this experiment, the SNR for each subcarrier is calculated using this method, as described in ITU-T G.9960. Specifically, the SNR of subcarrier *k* is expressed as

(5) $${\mathrm{SNR}}_{k}=\frac{P_{\mathrm{signal},k}}{P_{\mathrm{noise},k}}$$

where *P*_signal,*k*_ represents the received signal power of subcarrier *k* and *P*_noise,*k*_ represents the noise power for subcarrier *k*. The process of calculating the SNR for each subcarrier involves the following steps. First, the received signal power for each subcarrier is measured using the fast Fourier transform method, which enables precise evaluation of the signal’s strength in the frequency domain. Next, the noise power for each subcarrier is measured by estimating the baseline noise or error level in the absence of a signal, providing an accurate representation of the noise affecting the subcarrier. Finally, the ratio of the received signal power to the noise power is calculated for each subcarrier, allowing for a detailed assessment of communication quality across all subcarriers.

Remote devices without communication capabilities require specific methods to inform them of the local QAM format. Embedding the QAM format information in the PHY frame header enables receiving devices to decode the signal accordingly. Another method involves transmitting broadcast messages using a low-order QAM format, allowing remote devices to receive the information. Additionally, remote devices can be pre-configured with a predefined set of QAM formats, enabling the prediction of the format used under specific environmental conditions. These approaches ensure that devices without direct communication capabilities can adapt to the appropriate QAM format.

This method is employed in the current experiment to evaluate the communication quality of each subcarrier, enabling the optimization of adaptive modulation and error rate control based on the calculated SNR values.

## 5. Principle Experimental Results

**Figure 8:**
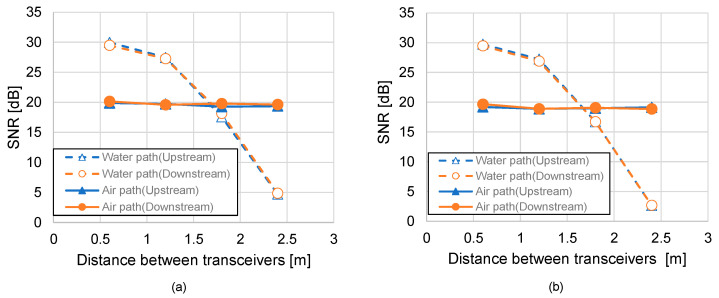
Static SNR characteristics of (**a**) tap water, (**b**) shallow seawater.

Figure 8a,b illustrate the static SNR characteristics for tap water and shallow seawater, respectively. The observed exponential decrease in the SNR for the underwater optical paths as the transmission distance increases highlights the significant impact of water turbidity and absorption on signal quality. In contrast, the air optical path demonstrates remarkable stability, with negligible variation in the SNR regardless of the distance. This finding emphasizes the effectiveness of switching to aerial paths when the underwater path experiences significant attenuation—switching from the underwater optical path to the air at a transceiver distance of 1.8 m yields better performance characteristics. Additionally, a comparison between tap water and shallow seawater reveals lower SNR values in seawater due to its higher turbidity and more substantial scattering effects. These results suggest that integrating real-time turbidity measurements into the system could further optimize the thresholds for switching between optical paths, thereby enhancing communication reliability.

**Figure 9:**
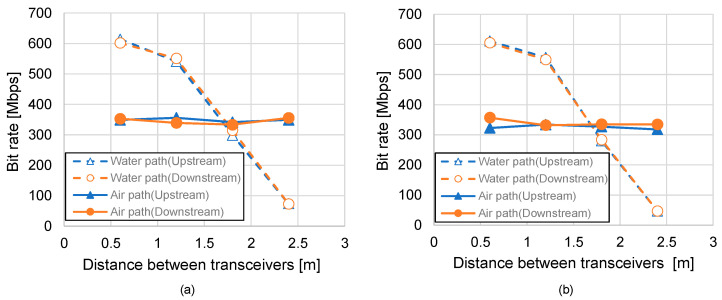
Static bit rate characteristics of (**a**) tap water, (**b**) shallow seawater.

Figure 9a,b show the static bit rate characteristics for tap water and shallow seawater under varying transmission distances. Like SNR trends, the bit rate for the underwater path declines exponentially with increasing distance, underlining the importance of adaptive modulation techniques to maximize data throughput. On the other hand, the aerial path maintains a nearly constant bit rate, demonstrating its stability in various conditions, and switching from the underwater optical path to the air at a transceiver distance of 1.8 m yields better performance characteristics here as well, confirming the superiority of the aerial path in overcoming underwater limitations. The performance gap between tap water and seawater further highlights the challenges of more turbid environments, where underwater communication performance deteriorates more rapidly. These findings underscore the system’s capability to prioritize aerial paths to maintain consistent communication quality, especially in scenarios with significant variations in underwater turbidity.

**Figure 10:**
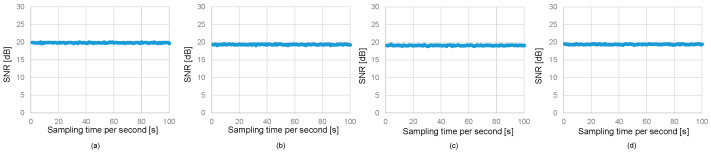
Dynamic SNR characteristics in uplink for (**a**) 0.6 m, (**b**) 1.2 m, (**c**) 1.8 m, (**d**) 2.4 m.

Figure 10a–d illustrate the dynamic SNR characteristics over 100 s in a shallow water channel under the same conditions as Figure 8, where the transceiver distance in the aerial path varies from 0.6 m to 2.4 m in increments of 0.6 m. The 5σ values are 0.43, 0.45, 0.44, and 0.57, respectively. The relatively stable SNR values in the underwater channel across these variations suggest that the short-term fluctuations in water quality have a minimal impact on communication performance.

**Figure 11:**
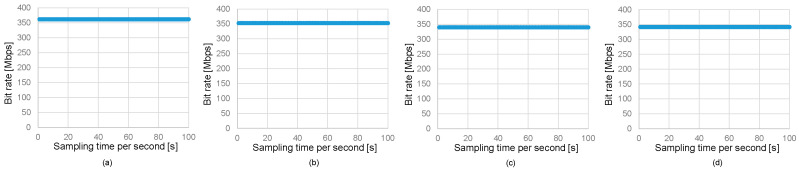
Dynamic bit rate characteristics in uplink for (**a**) 0.6 m, (**b**) 1.2 m, (**c**) 1.8 m, (**d**) 2.4 m.

Figure 11a–d show the dynamic bit rate characteristics over 100 s in a shallow water channel under the same conditions as Figure 9, where the transceiver distance in the aerial path varies from 0.6 m to 2.4 m in increments of 0.6 m. The stability of the bit rate demonstrates the robustness of the system’s adaptive beam steering mechanism, ensuring reliable alignment and data transmission.

## 6. Emulated Optical Path Switching Experiment for 4K Video Transmission

**Figure 12:**
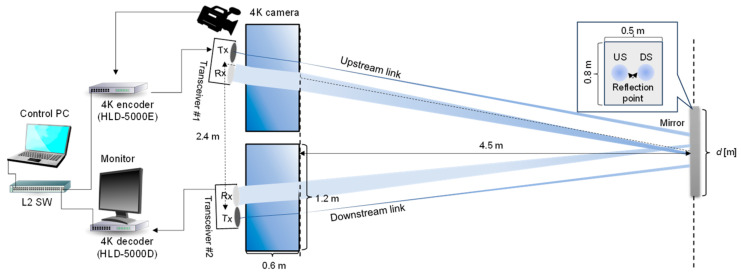
Experimental setup of 4K UHD video stream transmission.

Figure 12 illustrates the experimental setup for 4K UHD video stream transmission in a full-duplex optical wireless communication system emulating underwater/air optical path switching. The conditions for the air and underwater channels, optical transceivers, and mirrors in this experiment are the same as described in Section 3.

In the uplink transmission from Transceiver #1 to Transceiver #2, 4K video streaming is performed. Video captured by a 4K camera is input into a 4K encoder (HLD-5000E, IBEX Technology, Kanagawa, Japan), and the encoded video signal is sent to the transmitter of Transceiver #1. The optical signal passes through the underwater channel and is then transmitted via the air channel, where the receiver of Transceiver #2 receives it. The output signal is subsequently input into a 4K decoder (HLD-5000D, IBEX Technology, Kanagawa, Japan) and finally displayed on a monitor as high-resolution video. When the HLD-5000E and HLD-5000D are used as a paired codec, the latency is 20 ms, enabling near real-time video playback.

**Figure 13:**
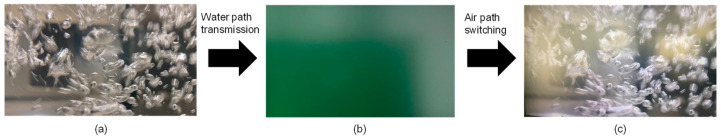
A 4K video image of (**a**) original, (**b**) underwater optical path, (**c**) aerial optical path.

Figure 13a shows the image quality of the 4K video before transmission, which serves as the baseline reference. However, as shown in Figure 13b, the image quality degrades significantly when the video is transmitted via the underwater optical path at a terminal distance of 2.4 m. This degradation is primarily caused by data loss, resulting in noticeable interference that current methods struggle to suppress completely. The receiver side’s frame reconstruction techniques are being considered to avoid this interference. In particular, video frame interpolation techniques utilizing profound learning show promise for achieving high-precision reconstruction, even in significant interference. On the other hand, as demonstrated in Figure 13c, high-quality video transmission can be achieved by switching the optical path to the air channel, where interference is minimized. The evaluation in this study is limited to a practical video transmission system designed for a specific transmission rate, emphasizing the importance of further advancements to ensure robust performance under diverse conditions.

## 7. Experiment on Steering Angle Misalignment Tolerance

**Figure 14:**
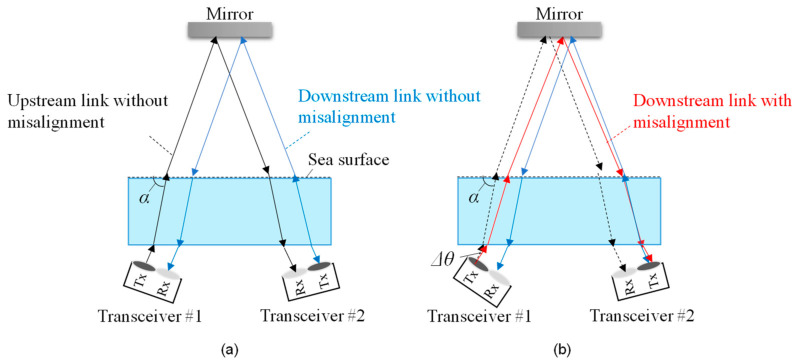
Experimental setup for evaluating resistance to steering misalignment: (**a**) without misalignment, (**b**) with misalignment.

Figure 14 illustrates the configuration of the experimental setup used to evaluate the impact of angular misalignment on one of the optical wireless transceivers. The two optical wireless transceivers are positioned simultaneously in this setup, with no positional displacement. In this experiment, angular misalignment Δ*θ* in the air channel is introduced up to a maximum of 2 degrees. Figure 14a shows the beam trajectory between the optical wireless transceivers under no angular misalignment. Their respective optical receivers successfully receive the beams emitted from each optical transmitter.

Figure 14b illustrates the beam trajectory when angular misalignment is introduced to one of the optical transceivers. The misaligned beam deviates from the intended trajectory due to changes in the reflection point on the mirror, causing the receiver to fail to capture the beam accurately.

**Figure 15:**
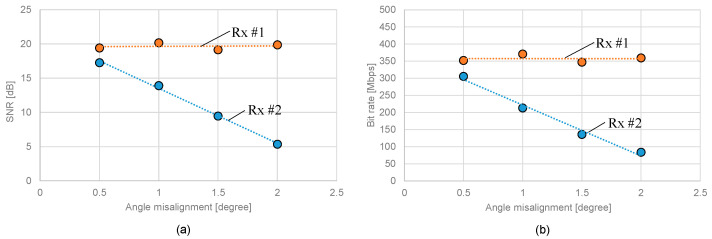
Tolerance to angular misalignment of (**a**) SNR and (**b**) bit rate.

Figure 15a,b presents the changes in received SNR and transmission capacity relative to the transceiver’s angular misalignment, taking the optimal angle (which provides the maximum bidirectional performance) as a reference. The results indicate a rapid degradation in both transmission capacity and received SNR when angular misalignment is introduced. At the maximum angular misalignment of 2 degrees, a reduction of 222 Mbps in transmission capacity and 12 dB in SNR was observed compared to the optimal angle.

## 8. Conclusions

This study demonstrated the feasibility and effectiveness of a bidirectional underwater optical wireless communication system capable of seamlessly switching optical paths between underwater and air environments according to environmental conditions. The proposed system leverages adaptive modulation and beam steering technologies to achieve high reliability and efficiency, even in underwater environments with high turbidity and long transmission distances.

The experimental results confirmed the system’s ability to achieve stable 4K UHD video streaming with low latency under aerial optical path conditions. Switching to the aerial optical path at a transceiver distance of 1.8 m significantly improved performance, overcoming the exponential signal degradation observed in underwater paths. At a distance of 2.4 m in shallow seawater, the system demonstrated a notable SNR improvement of 12 dB and maintained stable communication quality when switching to the aerial path.

However, the proposed system has its limitations. When the depthwise distance increases or the turbidity in the depthwise direction becomes more severe, losses in the aerial path can occur, making it difficult to achieve the required SNR for stable communication. These limitations highlight areas for further refinement of the system.

These findings underscore the potential to overcome the limitations of conventional underwater communication and establish a novel communication infrastructure for shallow water areas, remote locations, and disaster scenarios. Future challenges include supporting multi-node networks and optimizing performance under more complex environmental conditions.

## Figures and Tables

**Table 1:** Characteristics comparison of two relay types.

	O/E/O Relay	Mirror-Based Relay
Cost	High	Low
Weight	Heavy	Light
Power consumption	High	Low
Power budget	Large	Small
Alignment	Difficult	Easy

## Data Availability

The original contributions presented in the study are included in the article; further inquiries can be directed to the corresponding author.
